# Molecular mechanisms of pathogenicity of *Mycoplasma mycoides* subsp. *mycoides* SC

**DOI:** 10.1016/j.tvjl.2006.10.016

**Published:** 2007-11

**Authors:** Paola Pilo, Joachim Frey, Edy M. Vilei

**Affiliations:** Institute of Veterinary Bacteriology, University of Bern, Langgass-strasse 122, 3012 Bern, Switzerland

**Keywords:** *Mycoplasma mycoides* subsp. *mycoides* SC, CBPP, Pathogenicity, Capsular polysaccharide, Lipoproteins, Adhesion factors, Variable surface proteins, Toxic metabolic pathway products, Catabolite repression

## Abstract

*Mycoplasma mycoides* subsp. *mycoides* SC, the aetiological agent of contagious bovine pleuropneumonia (CBPP), is considered the most pathogenic of the *Mycoplasma* species. Its virulence is probably the result of a coordinated action of various components of an antigenically and functionally dynamic surface architecture. The different virulence attributes allow the pathogen to evade the host’s immune defence, adhere tightly to the host cell surface, persist and disseminate in the host causing mycoplasmaemia, efficiently import energetically valuable nutrients present in the environment, and release and simultaneously translocate toxic metabolic pathway products to the host cell where they cause cytotoxic effects that are known to induce inflammatory processes and disease. This strategy enables the mycoplasma to exploit the minimal genetic information in its small genome, not only to fulfil the basic functions for its replication but also to damage host cells in intimate proximity thereby acquiring the necessary bio-molecules, such as amino acids and nucleic acid precursors, for its own biosynthesis and survival.

## 1 Introduction

*Mycoplasma mycoides* subsp. *mycoides* small colony type (SC) is the aetiological agent of contagious bovine pleuropneumonia (CBPP), a severe infectious disease of cattle (Food and Agriculture Organization, 2003). *Mycoplasma* species are the smallest self-replicating organisms currently found on our planet. Their genomes range from 580 kb for *Mycoplasma genitalium* (Fraser et al., 1995) to 1358 kb for *Mycoplasma penetrans* (Sasaki et al., 2002), while that of *M. mycoides* subsp. *mycoides* SC has a size of 1211 kb (Westberg et al., 2004). This minimal genome has led the *Mycoplasma* species to radically economise genetic resources and biosynthetic capacities, and adapt to an obligate parasitic lifestyle (Razin, 1997).

The minimal cellular genome of mycoplasmas also serves as a blueprint for the design of synthetic live organisms (Check, 2002). In contrast to other pathogenic bacteria, where virulence is determined mainly by toxins, cytolysins and invasins, no such typical primary virulence genes have been found on the genomes of the ten *Mycoplasmas* species that have been sequenced completely (Fraser et al., 1995; Himmelreich et al., 1996; Glass et al., 2000; Chambaud et al., 2001; Sasaki et al., 2002; Papazisi et al., 2003; Jaffe et al., 2004; Minion et al., 2004; Vasconcelos et al., 2005). Mycoplasmas seem rather to use intrinsic metabolic and catabolic functions to cause disease in the affected host and to ensure the microbe’s survival (Abu-Groun et al., 1994; Baseman and Tully, 1997; Razin, 1997; Vilei and Frey, 2001; Pilo et al., 2005). Analysis of data from a severe mycoplasmal pathogen, such as *M. mycoides* subsp. *mycoides* SC, is expected not only to unravel the mechanisms of pathogenicity which lead to CBPP, but may also be useful in the better understanding of other pathogenic *Mycoplasma* species.

CBPP is the only animal disease belonging to the A list of most severe infectious animal diseases (as defined by the World Organisation for Animal Health – Office International des Epizooties, OIE) caused by a bacterium as a transmissible agent. CBPP has the potential for very serious and rapid spread across national borders. It is currently responsible for major losses in livestock production in Africa and, therefore, has serious socio-economic consequences and is of major importance in the international trade of animals and animal products. In most other parts of the world, CBPP was eradicated using drastic policies of stamping out cattle in infected areas, control of cattle movement, and quarantine (Turner, 1954, 1959). The re-emerging outbreaks of CBPP in some European countries at the end of the last century demonstrated the constant threat of the disease to industrialized countries and required the re-introduction of expensive eradication measures (Egwu et al., 1996).

The basic requirements to control CBPP are highly efficient diagnostic tests that detect *M. mycoides* subsp. *mycoides* SC not only in symptomatic animals but also in asymptomatic carriers, together with vaccine strategies that are able to reduce the infectious pressure in infected areas by preventing the pathogen from achieving host infection, colonization, and multiplication (Thiaucourt et al., 1998; Thiaucourt, 2002). In this context, it is worth noting that the live vaccines currently used to control CBPP in different regions of Africa were derived from strain T1 of *M. mycoides* subsp. *mycoides* SC, which was developed in Tanzania in the 1950s. The efficacy of T1 vaccine strains have been evaluated on several occasions and they have shown advantages but also drawbacks (Hubschle et al., 2002). For instance, cattle that were infected experimentally with strain T1/44 (or that were in contact with T1/44-infected animals) developed severe lung lesions characteristic for CBPP (Mbulu et al., 2004). Molecular analyses confirmed the identity of re-isolated T1/44, therefore the observed pathologies were attributed to this particular vaccine strain. Furthermore, inactivated whole cell vaccines appeared to exacerbate the effects of CBPP in adult cattle; as a result there have been few challenges with inactivated vaccines (Nicholas et al., 2004).

To cause disease or to be virulent, *M. mycoides* subsp. *mycoides* SC possesses particular mechanisms to adhere to the specific host tissue, to evade the host’s immune defence, to enable persistence and dissemination in the infected animal, and to cause inflammation and disease signs through cytotoxicity. The loss of any of these mechanisms can lead to attenuation or loss of virulence. Hence, a detailed knowledge of the molecular mechanisms of pathogenicity of *M. mycoides* subsp. *mycoides* SC is both a prerequisite to develop accurate diagnostic methods that allow pathogen detection and differentiate it from closely related bacterial species residing within the host, and to design safe and efficient vaccines. While the total virulence of *M. mycoides* subsp. *mycoides* SC can only be determined by expensive experimental infections of cattle, which are difficult to justify ethically, the various individual molecular mechanisms that contribute to virulence, such as adherence, antigenic variation, impact of capsular polysaccharides to allow persistence and dissemination in the host, or cytotoxic activity, can be studied by means of diverse in vitro immunoassays, cellular studies, or experimental mouse infection assays.

The recently established sequence of the complete genome of *M. mycoides* subsp. *mycoides* SC did not reveal any primary virulence factors such as toxins or invasins (Westberg et al., 2004). Therefore, the molecular mechanisms of pathogenicity of *M. mycoides* subsp. *mycoides* SC, as well as of most other *Mycoplasma* species, are “hidden” and require a consideration of the particularities of metabolic pathways, surface antigens, and their regulation. The present review will give a short overview of the molecular mechanisms of pathogenicity that have been unravelled to date and discuss their impact on the virulence of *M. mycoides* subsp. *mycoides* SC. In spite of the host specificity of *M. mycoides* subsp. *mycoides* SC, many basic molecular mechanisms of virulence are also expected to be found in other *Mycoplasma* species. DNA sequence analysis has revealed that the genomes of mycoplasmas consist of a minimal set of genes encoding the essential functions of life, which are conserved in most species and subspecies.

## 2 Immunopathogenicity

The principal pathological consequence of *M. mycoides* subsp. *mycoides* SC infections is a massive inflammatory reaction mainly restricted to the lungs of the affected cattle, leading to death by respiratory distress caused by lung consolidation in up to 30% of cases (Provost et al., 1987; Food and Agriculture Organization, 2003). In most infected cattle, however, CBPP is manifested in a chronic form, which allows the host to recover from disease but to remain a potential carrier of the pathogen and so a threatening reservoir of *M. mycoides* subsp. *mycoides* SC (Provost et al., 1987; Food and Agriculture Organization, 2003).

Dedieu and collaborators (2005a) recently studied the difference between the acute and chronic progression of CBPP in cattle at the molecular level. The authors performed a kinetic analysis of the response of cattle to an infection by *M. mycoides* subsp. *mycoides* SC and showed that in animals that recovered from disease, the CD4 Th1-like T-cell response to *M. mycoides* subsp. *mycoides* SC was maintained over a long period (until slaughter of the animals in the experiment). In contrast, in animals that developed an acute disease, progression of the symptomatic CBPP was associated with a decreased ability of the peripheral blood mononuclear cells to produce interferon (Dedieu et al., 2005a). These data indicated that the susceptibility of cattle to infection by *M. mycoides* subsp. *mycoides* SC is associated with switching the immune response to a mode that allows dissemination of the pathogen and progression of immunopathologies.

In an in vitro study on the apoptotic effect of *M. mycoides* subsp. *mycoides* SC on bovine peripheral blood mononuclear cells, Dedieu et al. (2005b) demonstrated that viable *M. mycoides* subsp. *mycoides* SC could induce strong morphological changes in the mononuclear cells, as evidenced by a reduction of cell size and increase of cell granularity, while heat-killed *M. mycoides* subsp. *mycoides* SC had no effect. This cytopathic effect was shown to be the cause of apoptosis of the mononuclear cells triggered by live *M. mycoides* subsp. *mycoides* SC or by a substance released by mycoplasmas. However, the triggering effect measured by mycoplasma free culture supernatants was rather low (Dedieu et al., 2005b), suggesting that the triggering agent has a short half-life or acts only over very short distances.

Several other reports suggest that potentially secreted substances of *M. mycoides* subsp. *mycoides* SC may be the cause of the immunopathological effects of CBPP. However, the results of genomic DNA sequence analysis of *M. mycoides* subsp. *mycoides* SC (Westberg et al., 2004), as well as genome sequences of other *Mycoplasma* species, did not reveal any particular candidate molecules that could be directly identified as molecular triggers for apoptosis or cell necrosis.

## 3 Secreted polycarbohydrates and capsular polysaccharide

**Fig. 1:**
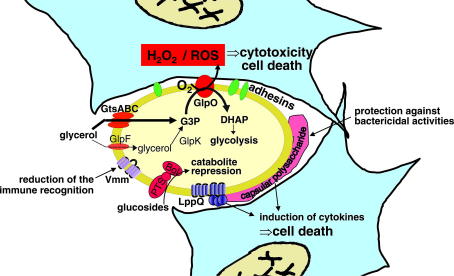
Schematic representation of the various virulence pathways of *M. mycoides* subsp. *mycoides* SC. The mycoplasma is represented in yellow, the host cells in light blue. The upper pathway, which is currently the best characterized, is based on glycerol import and metabolism. GtsABC represents the membrane-located ATP-binding cassette (ABC) transporter system, which incorporates and phosphorylates imported glycerol to glycerol-3-phosphate (G3P). The bypass pathway that allows diffusion of glycerol via the glycerol facilitator factor (GlpF) and the subsequent phosphorylation by the glycerol kinase (GlpK) has not been evidenced directly, but the corresponding genes, *glpF* and *glpK*, have been found by cloning and DNA sequence analysis, and their functionality was supported by strains lacking functional *gtsABC* genes, which can import and phosphorylate glycerol. The membrane-located l-α-glycerophosphate oxidase (GlpO) represents the central enzyme in this virulence pathway and is able to translocate H_2_O_2_ or reactive oxygen species (ROS) into the host cell. The adhesins that seem necessary for the close contact are still hypothetical. The variable surface antigen (Vmm) is supposed to play a role in evading the host’s immune defence. Capsular polysaccharide has been proposed to be produced by most *Mycoplasma* species and is expected to induce cytokine production. Lipoprotein LppQ is highly antigenic and also seems to be involved in the induction of inflammatory processes by superantigen-like properties. Catabolism of glucosides by the phosphotransferase system (PTS) and 6-phospho-β-glucosidase (Bgl) is known to repress virulence of many pathogens.

Molecules located on the outer surface or in the plasma membrane of *Mycoplasma* species, such as lipoproteins, capsular polysaccharides and biofilms composed mainly of fibrillar polycarbohydrates surrounding cells, are generally assumed to protect the pathogen from the bactericidal activity of complement and other host defence functions, and to trigger the inflammatory process in the infected host (Fig. 1). Biofilms lead bacteria to adhere among themselves and to form micro-colonies on mucosal surfaces and inert materials that are mostly resistant to bactericidal activity of the host and to antibiotics. However, *M. mycoides* subsp. *mycoides* SC does not form biofilms as shown in a recently published study, where 25 strains from different origins and with variable degrees of virulence were tested for biofilm production (McAuliffe et al., 2006).

The capsular polysaccharide galactan, composed of 6-*O*-β-d-galactofuranosyl-d-galactose (Plackett and Buttery, 1964), was shown to increase the virulence of the strongly attenuated *M. mycoides* subsp. *mycoides* SC vaccine strain KH_3_J when added to the inoculum prior to experimental infections of cattle (Hudson et al., 1967). Moreover, Buttery et al. (1976) showed that intravenous injection of galactan from *M. mycoides* subsp. *mycoides* SC to calves produced transient apnoea, increased pulmonary arterial pressure and pulmonary oedema, indicating that capsular polysaccharide of this species has a direct cytopathic effect and seems to lead to the contraction of blood vessels, which may initiate thrombosis. The impact of capsular polysaccharide on virulence was also shown by growth inhibition tests, which primarily measure serum resistance, and by a mouse infection model, which mainly measures the capacity of the *Mycoplasma* strain to cause bacteraemia.

March et al. (1999) showed that a strain of *M. mycoides* subsp. *mycoides* SC that produces low amounts of capsular polysaccharide was much more sensitive to growth inhibiting antisera than strains that produced larger amounts of polysaccharide. Furthermore, the same strains that produced large amounts of capsular polysaccharide also generated a significantly longer duration of bacteraemia in a mouse infection assay than the strain with little capsular polysaccharide (March and Brodlie, 2000). Hence, capsular polysaccharide seems to play a role in the capacity of persistence and dissemination of *M. mycoides* subsp. *mycoides* SC in the infected host (Fig. 1). This process is independent of the strain’s capacity to release H_2_O_2_ upon glycerol uptake and metabolism, a mechanism that affects the cytotoxicity of *M. mycoides* subsp. *mycoides* SC (Vilei and Frey, 2001; Pilo et al., 2005). For this reason, the virulence (as measured using the mouse infection assay) and the ability of the different strains of *M. mycoides* subsp. *mycoides* SC to metabolize glycerol were assumed to be unconnected, as reported previously (March and Brodlie, 2000).

## 4 Surface proteins

Lipoproteins are expected to play a role as triggers in mechanisms of pathogenicity, since they are known to have a central role in interactions between mycoplasmas and eukaryotic cells, particularly with respect to adhesion. Furthermore, lipoproteins have been reported to stimulate the release of pro-inflammatory cytokines (Mühlradt and Frisch, 1994; Herbelin et al., 1994; Brenner et al., 1997; Marie et al., 1999; Calcutt et al., 1999). Since lipoproteins are usually strongly antigenic proteins, they are also considered to be valuable targets for specific and sensitive serodiagnosis. Currently, a few lipoproteins from *M. mycoides* subsp. *mycoides* SC have been characterized in detail. Most of them are major antigens and are readily detected in the serum of infected cattle on immunoblots.

LppA is strongly conserved among mycoplasmas of the “*M. mycoides* cluster” and it is expressed by highly virulent *Mycoplasma* species as well as by non-pathogenic or low-pathogenic species (Monnerat et al., 1999a; Monnerat et al., 1999b). The role of LppA in Th1 and Th2 immunity is currently under investigation. LppB is found only in strains belonging to the African/Australian cluster, but is not found in strains isolated from the re-emerging European outbreaks in 1980–1999. LppB is, however, also present in other mycoplasmas of the “*M. mycoides* cluster”, in particular, in the bovine pathogen *Mycoplasma* sp. bovine group 7 (Vilei et al., 2000). Another lipoprotein, LppC, which is expressed by all *M. mycoides* subsp. *mycoides* SC strains, is also found in other, less pathogenic *Mycoplasma* species (Pilo et al., 2003). Hence, the role of these lipoproteins in virulence is still unclear. LppQ is a lipoprotein specific to *M. mycoides* subsp. *mycoides* SC. It has a particularly strong antigenic N-terminal domain located on the outer surface of the membrane, while its C-terminal domain is involved in membrane anchoring (Abdo et al., 2000).

The high specificity and strong antigenicity of LppQ have been exploited for the development of a robust indirect ELISA test for serological diagnosis and for epidemiological investigations of CBPP (Bruderer et al., 2002). Structural analysis of LppQ showed strong similarities to proteins with super-antigenic character (Abdo et al., 2000). A recent study has shown that cattle immunized with purified recombinant LppQ, using different adjuvant methods, were significantly more susceptible to challenge with *M. mycoides* subsp. *mycoides* SC than cattle that were not vaccinated with LppQ (Nicholas et al., 2004). Hence, LppQ is assumed to play an adverse reaction in vaccination, similar to the peptidoglycan-associated lipoprotein PalA of *Actinobacillus pleuropneumoniae*, which was shown to completely inhibit the beneficial effects of efficient subunit vaccines when animals were vaccinated simultaneously with PalA (van den Bosch and Frey, 2003). LppQ may therefore contribute to the immunopathologies induced by *M. mycoides* subsp. *mycoides* SC (Fig. 1).

## 5 Adhesion factors

Adhesins play an important role in the early steps of pathogenesis of most microorganisms. Since mycoplasmas do not seem to secrete toxins that could act over long distances, adhesion is particularly important in mycoplasmal virulence. Adhesion plays a central role in the intimate interactions of pathogenic mycoplasmas with mammalian cells for long periods and is assumed to trigger a cascade of signals, which are transduced to the host cell and induce inflammation (Razin et al., 1998). Adhesins also seem to be responsible for host specificity and tissue tropism. Although several adhesins have been identified in various *Mycoplasma* species (Sachse et al., 1996; Noormohammadi et al., 1997; Razin et al., 1998; Kitzerow et al., 1999; Sachse et al., 2000; Minion et al., 2000; Fleury et al., 2002; Belloy et al., 2003), specific adhesins have not yet been detected in *M. mycoides* subsp. *mycoides* SC, but can be postulated to occur from the above considerations.

## 6 Variable surface proteins

Phase variation is a common mechanism among *Mycoplasma* species and is thought to be involved in microbial survival, leading to the emergence of varied intra-clonal populations that adapt quickly to new environments (Dybvig and Voelker, 1996; Henderson et al., 1999). Some *Mycoplasma* species possess an abundance of phase-variable proteins, especially surface-exposed lipoproteins (Citti and Wise, 1995; Citti et al., 2000; Röske et al., 2001; Denison et al., 2005).

A variable surface protein, designated Vmm, has been discovered in *M. mycoides* subsp. *mycoides* SC (Persson et al., 2002). Vmm is a 16-kDa protein, specific to this *Mycoplasma* species. It is expressed by nearly all strains analyzed, where it has shown a reversible ON–OFF phase variation at a high frequency per cell generation. This variation is regulated at the transcriptional level by dinucleotide insertions or deletions in a repetitive region of the promoter (Persson et al., 2002). Genes resembling the *vmm* gene were also found in other species of mycoplasma, but analogous Vmm-like proteins in these species could not be detected with a specific monoclonal antibody directed to Vmm of *M. mycoides* subsp. *mycoides* SC. The function of Vmm is currently unknown, but repeating elements in variable membrane proteins of mycoplasmas have been suggested to increase the pathogen’s ability to adhere to host cells and to evade the host immune response (Peterson et al., 1995; Sachse et al., 2000) (Fig. 1).

## 7 Toxic metabolic pathway products

Oxygen uptake and H_2_O_2_ production were identified as particular characteristics in fermentative *Mycoplasma* species and were expected to influence the virulence of pathogenic mycoplasmas (Cole et al., 1968; Brennan and Feinstein, 1969; Wadher et al., 1990; Miles et al., 1991). It was later discovered that *M. mycoides* subsp. *mycoides* SC strains isolated from the re-emerging European outbreaks of CBPP in 1980–1999 produced much less H_2_O_2_ when grown in the presence of glycerol than strains isolated in the African and Australian continents and it was suggested that a glycerophosphate oxidase could represent a significant virulence factor of *M. mycoides* subsp. *mycoides* SC (Wadher et al., 1990; Houshaymi et al., 1997; Rice et al., 2001).

Recently, the role of glycerol metabolism in the virulence of *M. mycoides* subsp. *mycoides* SC was also considered in our laboratory, based on the observation that African strains contain an efficient active glycerol import system, GtsABC, specified by an ABC transporter protein, while the European strains, which are considered less virulent (Abdo et al., 1998) and cause a disease that appears to be largely chronic showing few clinical signs and low mortality (Nicholas et al., 1996), are devoid of this transporter protein (Vilei and Frey, 2001). Glycerol is metabolized after uptake and phosphorylation to dihydroxyacetone phosphate (DHAP) by an oxidative process leading to the release of the highly toxic compound H_2_O_2_. Blocking the glycerol uptake proteins GtsABC by specific antibodies resulted in a significant reduction of H_2_O_2_ production (Vilei and Frey, 2001). Furthermore, it was shown that European strains that lack the GtsABC transporter produce significantly lower amounts of H_2_O_2_.

The l-α-glycerophosphate oxidase (GlpO), which catalyzes the oxidation of glycerol-3-phosphate (G3P) accompanied by the release of a molecule of H_2_O_2_, was identified as the membrane protein that plays a central role in cytotoxicity of *M. mycoides* subsp. *mycoides* SC strains towards embryonic calf nasal epithelial (ECaNEp) cells (Pilo et al., 2005). In the presence of physiological concentrations of glycerol, relatively large amounts of H_2_O_2_ were released into the culture medium. When ECaNEp cells were exposed to African *M. mycoides* subsp. *mycoides* SC field strains in the presence of physiological concentrations of glycerol, H_2_O_2_ was rapidly detected in their cytosol and, subsequently, cell death occurred. Only a weak cytotoxic effect was observed when ECaNEp cells were infected with European strains. Culture supernatants from *M. mycoides* subsp. *mycoides* SC grown in the presence of glycerol, and thus containing up to 150 μM H_2_O_2_, or buffers or axenic culture media supplemented with 150 μM H_2_O_2_ had no cytotoxic effect on ECaNEp cells. In these cases, H_2_O_2_ or the accompanying reactive oxygen species (ROS) could not be detected in the cytosol of the eukaryotic cells (Pilo et al., 2005). Hence, close contact between mycoplasmas and host cells was necessary in order to successfully translocate the toxic compounds from the glycerol metabolism into the host cells. In this context, it is worth noting that most *Mycoplasma* species, including *M. mycoides* subsp. *mycoides* SC, remain tightly attached to the surface of epithelial cells but do not penetrate them (for review see Razin et al., 1998). A possible mechanism of adhesion, particularly in calf epithelial cells, could be the partial fusion of the surface exposed epitopes of *M. mycoides* subsp. *mycoides* SC with the host cell wall (Pilo et al., 2005).

A proposed model for triggering cellular damage to eukaryotic cells is that glycerol present in the interstitial fluid is incorporated actively via the highly active ABC glycerol transporter GtsABC and is subsequently phosphorylated into G3P. This, in turn, is oxidized by GlpO into DHAP, which enters in the glycolysis cycle of the mycoplasma, and H_2_O_2_ is released. Facilitated by the intimate contact of the mycoplasma with the host cell membrane, H_2_O_2_ or ROS enters the host cell. Inside the host cells, these toxic compounds act as powerful mediators of cell injury and inducers of inflammatory processes (Fig. 1). They are expected to damage the host either by directly impairing tissue cells or by inducing host gene expression, e.g. of pro-inflammatory genes via activation of the nuclear factor kappa B (NF-κB) (Baeuerle and Henkel, 1994), or via the Fenton reaction (Crichton et al., 2002).

Interestingly, mycoplasmas have previously been shown to induce a respiratory burst in phagocyte cells (Koppel et al., 1984), suggesting that host-generated ROS might further contribute to tissue damage. Furthermore, it was shown that *M. mycoides* subsp. *mycoides* SC strains are also able to induce the tumor necrosis factor alpha (TNF-α) in bovine alveolar macrophages (Jungi et al., 1996). TNF-α also acts as an inflammatory mediator and, in association with NF-κB, can act as primary signals inducing apoptosis in host cells. The demonstration of the involvement of these two molecules in an apoptotic pathway triggered by *M. mycoides* subsp. *mycoides* SC in host cells must be investigated.

## 8 Catabolite repression of bacterial virulence

A non-synonymous single nucleotide polymorphism (SNP) in the *bgl* gene encoding the 6-phospho-β-glucosidase (Bgl) has recently been detected. It differentiates African field strains from European field strains from the 1980–1999 outbreaks, as well as currently used vaccine strains of *M. mycoides* subsp. *mycoides* SC (Vilei and Frey, 2004). Bgl is an intracellular enzyme associated with the phosphoenolpyruvate-dependent sugar:phosphotransferase system (PEP–PTS), a multicomponent system involved in the simultaneous translocation and phosphorylation of sugars by bacteria from both Gram-positive and Gram-negative genera. Sugars in general and sugar catabolites are known to mediate repression of bacterial virulence gene expression; in particular, catabolism of β-glucosides is involved in the regulation of the virulence of bacterial pathogens, such as *Listeria monocytogenes* (Brehm et al., 1999) and *Erwinia amylovora* (Kerppola et al., 1987). Hence, it will be worthwhile in the future to analyze the role of the *bgl* gene in attenuated strains of *M. mycoides* subsp. *mycoides* SC, and to investigate whether the resulting enzyme shows a regulated activity that would be able to catabolically modulate the mycoplasmal virulence characteristics.

## 9 Concluding remarks

The pathogenicity of *M. mycoides* subsp. *mycoides* SC seems to be multifunctional, as would be expected from knowledge of the molecular mechanisms of pathogenicity from other bacterial species. However, in contrast to the latter, whose virulence is determined by special virulence factors such as toxins and invasins, the virulence factors of *Mycoplasma* species seem to be determined by intrinsic metabolic or catabolic pathway functions or by proper constituents of the mycoplasmal outer surface. In *M. mycoides* subsp. *mycoides* SC, a few virulence determinants have been unravelled in recent years including capsular polysaccharide, which seems to be involved in serum-resistance to give the organism the capacity to persist and disseminate in the host. In addition, there are unknown but necessary adhesion factors, immunomodulating factors, and toxic metabolic pathway products, which exert cytotoxic effects and can induce inflammatory reactions in the host. All these virulence mechanisms are measured by various in vitro or biological assays.

The loss of any of these virulence mechanisms can lead to attenuation or, potentially, to avirulence, even when all the other virulence attributes are still fully active. This is well known for many other bacterial pathogens. For example, in *Bacillus anthracis*, the loss of the capsule biosynthesis genes on plasmid pXO2 leads to an avirulent strain. The latter is currently used as a live vaccine, in spite of the presence of the oedema factor and the lethal toxin (Brossier and Mock, 2001). It is therefore not surprising that *M. mycoides* subsp. *mycoides* SC strains, such as KH_3_J, that produce low amounts of capsular polysaccharide and have lost most of their potential to cause bacteraemia in mice but still produce large amounts of H_2_O_2_ by their capacity to import and metabolize glycerol, have a strongly attenuated virulence (Lloyd et al., 1971; Dyson and Smith, 1976; March et al., 1999; March and Brodlie, 2000; Rice et al., 2001).

The various approaches and methods to study virulence of *M. mycoides* subsp. *mycoides* SC target individual mechanisms. To assess the overall virulence of *M. mycoides* subsp. *mycoides* SC, all of these different mechanisms of pathogenicity would have to be considered. Currently, a major handicap in the study of the basic principles of the different virulence mechanisms of *M. mycoides* subsp. *mycoides* SC is the lack of a genetic basis for most of them. In order for research on the molecular mechanisms of pathogenicity of *M. mycoides* subsp. *mycoides* SC to progress, efficient tools for targeted mutagenesis and vectors able to trans-complement mutations, as have been constructed for other related *Mycoplasma* species (Janis et al., 2005), will be essential. Such methodologies will not only be useful to drive basic research on molecular pathogenicity, but will also be valuable for the targeted construction of novel, efficient, and safe vaccines against CBPP in areas where the disease is endemic. In fact, vaccines currently administered to cattle are in the form of live bacteria and pose a potential threat to livestock especially in areas so far free of CBPP. Their biological safety aspects require particular attention by implementing safe and non-reversible attenuation sites in new vaccine strains.
